# Supplementary material for: Resolving the full spectrum of human genome variation using Linked-Reads
Source: Genome Res. 2019 Apr;29(4):635–45. doi: 10.1101/gr.234443.118 (PMC6442396; doi:10.1101/gr.234443.118)
Supplement: Supplemental Material [file supp_gr.234443.118_Supplemental_longranger-2.2.zip › longranger-2.2.tar.bz2!/lr2.2-public/lib/stan/cmdstan/stan/doc/api/html/expression07__grammar__def_8hpp.html]

Stan: src/stan/gm/grammars/expression07\_grammar\_def.hpp File Reference


|  |  |
| --- | --- |
|  | Stan  2.5.0  probability, sampling & optimization |


- Main Page
- Related Pages
- Namespaces
- Classes
- Files

- File List
- File Members

All Classes Namespaces Files Functions Variables Typedefs Enumerations Enumerator Friends Macros Pages

- src
- stan
- gm
- grammars

Classes |
Namespaces |
Variables

expression07\_grammar\_def.hpp File Reference

`#include <cstddef>`  
`#include <iomanip>`  
`#include <iostream>`  
`#include <istream>`  
`#include <map>`  
`#include <set>`  
`#include <sstream>`  
`#include <string>`  
`#include <utility>`  
`#include <vector>`  
`#include <stdexcept>`  
`#include <boost/spirit/include/qi.hpp>`  
`#include <boost/spirit/include/phoenix_core.hpp>`  
`#include <boost/spirit/include/phoenix_function.hpp>`  
`#include <boost/spirit/include/phoenix_fusion.hpp>`  
`#include <boost/spirit/include/phoenix_object.hpp>`  
`#include <boost/spirit/include/phoenix_operator.hpp>`  
`#include <boost/spirit/include/phoenix_stl.hpp>`  
`#include <boost/lexical_cast.hpp>`  
`#include <boost/fusion/include/adapt_struct.hpp>`  
`#include <boost/fusion/include/std_pair.hpp>`  
`#include <boost/config/warning_disable.hpp>`  
`#include <boost/spirit/include/qi_numeric.hpp>`  
`#include <boost/spirit/include/classic_position_iterator.hpp>`  
`#include <boost/spirit/include/support_multi_pass.hpp>`  
`#include <boost/tuple/tuple.hpp>`  
`#include <boost/variant/apply_visitor.hpp>`  
`#include <boost/variant/recursive_variant.hpp>`  
`#include <stan/gm/ast.hpp>`  
`#include <stan/gm/grammars/whitespace_grammar.hpp>`  
`#include <stan/gm/grammars/term_grammar.hpp>`  
`#include <stan/gm/grammars/expression_grammar.hpp>`  
`#include <stan/gm/grammars/expression07_grammar.hpp>`

Go to the source code of this file.

|  |  |
| --- | --- |
| Classes | |
| struct | stan::gm::validate\_expr\_type3 |
|  | |
| struct | stan::gm::validate\_expr\_type3::result< T1, T2 > |
|  | |
| struct | stan::gm::set\_fun\_type3 |
|  | |
| struct | stan::gm::set\_fun\_type3::result< T1, T2 > |
|  | |
| struct | stan::gm::addition\_expr3 |
|  | |
| struct | stan::gm::addition\_expr3::result< T1, T2, T3 > |
|  | |
| struct | stan::gm::subtraction\_expr3 |
|  | |
| struct | stan::gm::subtraction\_expr3::result< T1, T2, T3 > |
|  | |

|  |  |
| --- | --- |
| Namespaces | |
|  | stan |
|  | Probability, optimization and sampling library. |
|  | |
|  | stan::gm |
|  | Directed graphical model compiler. |
|  | |

|  |  |
| --- | --- |
| Variables | |
| boost::phoenix::function  < validate\_expr\_type3 > | stan::gm::validate\_expr\_type3\_f |
|  | |
| boost::phoenix::function  < set\_fun\_type3 > | stan::gm::set\_fun\_type3\_f |
|  | |
| boost::phoenix::function  < addition\_expr3 > | stan::gm::addition3\_f |
|  | |
| boost::phoenix::function  < subtraction\_expr3 > | stan::gm::subtraction3\_f |
|  | |


---

[ Stan Home Page ]

*© 2011–2014,
Stan Development Team.*
